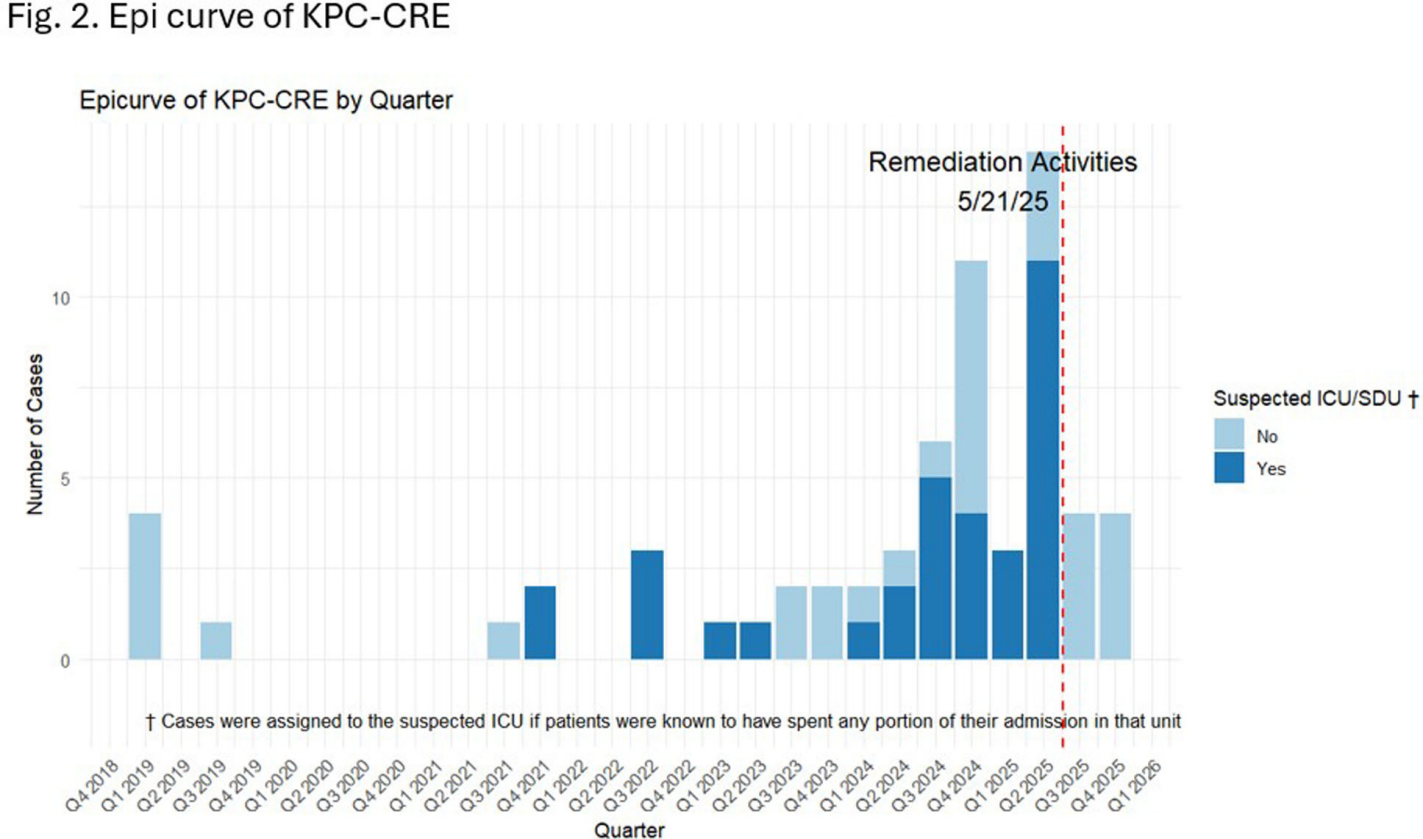# 330 A multifaceted approach to reducing reportable central-line-associated bloodstream infections

**DOI:** 10.1017/ash.2026.10675

**Published:** 2026-06-23

**Authors:** Juan Teran Plasencia, Kelly Cawcutt, Maroya Walters, Miranda Neumann, Samuel Cincotta, Richard Stanton, Peter Iwen, Michael Wiley, Lacey Pavlovsky, Trevor Van Schooneveld, Jonathan Ryder, Joe Sexton, Heather Moulton-Meissner, Paige Gable, Christine Ganim, Alyssa Kent, Jennifer Vogelsberg, Emily Barg, Emily McCutchen, Paul D. Fey, Amanda Bartling, Amy Roden, Stevie McGill, Claire Ortlieb, M. Salman Ashraf

**Affiliations:** 1 University of Nebraska Medical Center; 2 Centers for Disease Control and Prevention; 3 Nebraska Medicine; 4 CDC; 5 Nebraska Public Health Laboratory; 6 Nebraska Department of Health and Human Services; 7 Centers for Disease Control

## Abstract

**Background:** After CDC and local surveillance identified an increase in Klebsiella pneumoniae carbapenemase (KPC)-producing carbapenem-resistant Enterobacterales (KPC-CRE) caused by multiple organisms, an investigation was conducted at an acute care hospital. Cases clustered around an intensive care unit (ICU) and step-down unit (SDU), where wastewater plumbing was a suspected transmission source. Whole genome sequencing (WGS) was used to investigate this outbreak. **Methods:** A case was defined as detection of KPC-CRE or KPC (no organism cultured) from specimens collected at the hospital between 01/2019 to 10/2025. Clinical cases were identified via carbapenemase testing of CRE isolates using PCR (Xpert Carba-R) or by multiplex PCR panels and screening cases through PCR with reflex to culture. ICU sink and hopper samples and SDU sink samples were tested using selective media, MALDI-TOF, and multiplex PCR for KPC-CRE detection. Isolates underwent short and long-read sequencing. **Result:** Overall, 64 cases (37 clinical; 27 screening), involving 54 patients, were identified; 28 patients had ICU/SDU admissions during their hospitalization. The 52 cases with an organism identified included seven species, of which the most common were Citrobacter (26), Klebsiella (14), and Raoultella (5); 24 harbored blaKPC-2 (37.5%), 18 blaKPC-4 (28.1%), 5 blaKPC-3 (7.8%), 1 blaKPC-160 (1.5%), and 16 were not subtyped (25%). Seventeen isolates from five species carried blaKPC-2 on a 42 kb IncP6 plasmid, while ten isolates from five species carried blaKPC-4 on a 69 kb plasmid with Col440I and IncM1 replicons. Environmental sampling in 05/2025 found KPC-CRE in 10/12 sinks and 2/12 hoppers in the ICU and 1/22 SDU sinks (fig1). KPC-CRE environmental isolates were closely genetically related to the Citrobacter, Klebsiella, and Roultella clinical isolates and harbored similar KPC-carrying plasmids. Infection prevention and control (IPC) rounding identified patient items within the sink splash zone, including placing bath-in-a-bag in the sink. In 05/2025, a campaign was initiated to remove items from the splash zone, switch to chlorhexidine wipes, and implement daily use of an EPA-registered, sodium dichloroisocyanurate-based biofilm drain disinfectant product. Starting 07/2025, pouring of lipids and total parenteral nutrition down drains was stopped. While repeat environmental sampling in 6/2025 also found KPC-CRE in sink drains, no additional cases were identified in the ICU or SDU following these remediation activities (fig2). **Conclusion:** ICU sinks served as a reservoir for KPC-CRE, with transmission to patients. KPC-CRE persisted in drains following initial drain disinfection measures; therefore, changes in sink and splash-zone IPC practices appear to be important for interrupting transmission.

**Figure:**
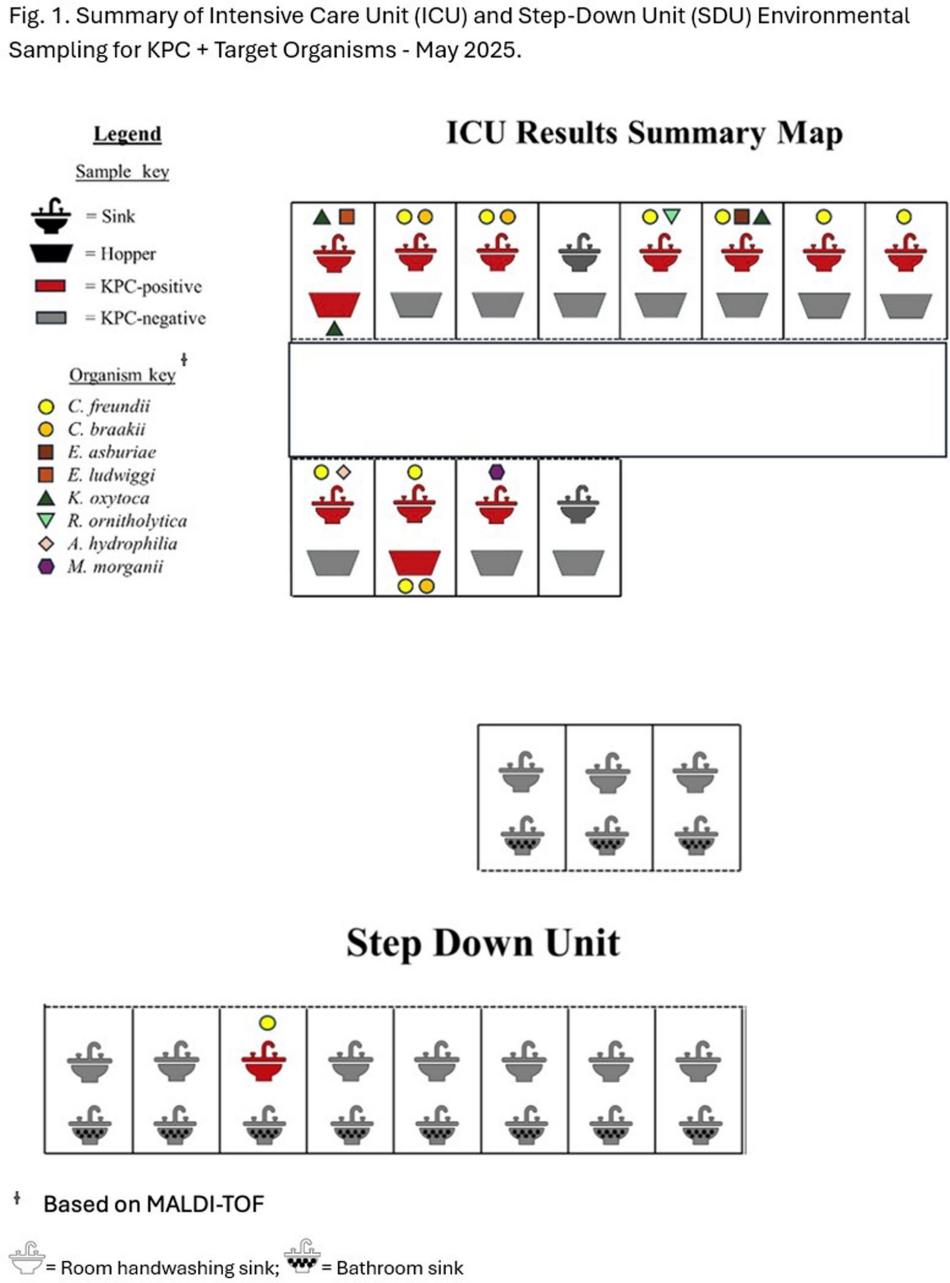

**Figure:**